# Chloroplastic protein prenylation: an internal or external affair? Reconciling biochemical data through a new hypothesis

**DOI:** 10.3389/fpls.2026.1873377

**Published:** 2026-08-19

**Authors:** Manfei Luo, Wei Chi

**Affiliations:** College of Life Sciences, Nanjing Normal University, Nanjing, China

**Keywords:** chloroplast, chloroplast protein importing, post-translational modifications, prenylation, TIC-TOC complex

## Abstract

Protein prenylation facilitates membrane association, protein interactions, and trafficking by attaching isoprenoid groups to C-terminal cysteine residues in the cytosol, with subsequent proteolysis and carboxymethylation occurring in the endoplasmic reticulum. In contrast this classic pathway, an organelle-autonomous prenylation pathway in plastids has long been hypothesized, however, its biochemical mechanisms remain unconfirmed. This review synthesizes multi-omics and genetic evidence to propose that plastid prenylation likely involves nuclear-encoded proteins modified in the cytosol, with final processing at the chloroplast envelope rather than the endoplasmic reticulum. In addition, the processing of prenylated chloroplast protein precursors may be integrated into the protein targeting machinery. By challenging the notion of fully chloroplast-independent prenylation, this hypothesis and theory article offers new insights into organellar protein modification in eukaryotes.

## Introduction

The functional diversity of the proteome extends far beyond what is encoded in genomic sequences, largely through post-translational modifications (PTMs) (Friso and van Wijk, 2015; Vu et al., 2018). These covalent chemical modifications of specific amino acid residues, which can be reversible or irreversible, dramatically expand protein functionality and enable rapid cellular adaptation at minimal metabolic cost (Friso and van Wijk, 2015; Vu et al., 2018). Among the various PTMs, covalent lipid attachment is a key mechanism for regulating protein localization and activity. By increasing hydrophobicity, lipid anchors enable soluble proteins to associate with membranes and can directly modulate protein function, as seen in ion channel proteins and signaling proteins (Thompson and Okuyama, 2000). In plants, hundreds of proteins undergo lipid modification, with one of the most significant being cysteine-linked prenylation, the attachment of either a 15-carbon farnesyl or a 20-carbon geranylgeranyl group (Galichet and Gruissem, 2003; Hemsley, 2015; Turnbull and Hemsley, 2017). Based on conserved C-terminal motifs, over 700 Arabidopsis proteins are predicted to be prenylated, implicating this modification in diverse processes including transcription, cell cycle regulation, cell wall dynamics, and stress responses (Galichet and Gruissem, 2003; Hemsley, 2015; Turnbull and Hemsley, 2017).

Despite this broad occurrence, the prenylation of one class of proteins, chloroplast proteins, remains poorly understood and somewhat paradoxical. This knowledge gap likely stems from the multi-step nature of prenylation, which involves not only lipid attachment but also subsequent proteolytic cleavage and methylation, events that typically occur in distinct cellular compartments (Galichet and Gruissem, 2003; Hemsley, 2015; Turnbull and Hemsley, 2017). Early studies suggested the existence of an autonomous prenylation pathway inside chloroplasts (Parmryd et al., 1997, but this hypothesis has received limited experimental support in recent decades.

Chloroplasts, as endosymbiotic organelles, retain their own genome but import the vast majority of their proteins from the cytosol through the coordinated TOC and TIC translocons at the envelope (Jarvis and López-Juez, 2013; Nakai, 2018). In this review, we integrate multi-omics and genetic evidence to propose a revised model: prenylation of chloroplast-targeted proteins likely begins in the cytosol, with final processing steps occurring at the chloroplast envelope itself rather than in the endoplasmic reticulum. Furthermore, we explore how prenylation may be functionally integrated into the chloroplast protein import machinery. By challenging the idea of a fully chloroplast-autonomous prenylation pathway, this synthesis offers a fresh perspective on the spatial organization of protein modification in eukaryotic cells.

## The classic protein prenylation pathway

Protein prenylation involves the formation of a stable thioether bond between a cysteine residue in the target protein and either a 15-carbon farnesyl or a 20-carbon geranylgeranyl isoprenoid group (Rilling et al., 1989; Boyartchuk et al., 1997; Rodriguez-Concepcion et al., 1999). Depending on the specific prenyltransferases and the subsequent maturation process, protein prenylation is classified into two types: Type I and Type II (**Table 1**). For type 1, this modification occurs via a three-step process: (1) isoprenoid attachment to a cysteine in a C-terminal prenylation motif, which is referred to “CaaX”, a three-amino acid motif where “aa” represents two aliphatic amino acids, and “X” represents the C-terminal amino acid that dictates the specificity of the modification, (2) proteolytic removal of the “aaX” residues, and (3) carboxy-terminal methylation. These coordinated events take place in the cytosol and on the endoplasmic reticulum (ER) (Rodriguez-Concepcion et al., 1999). Conversely, for Type II, the second and third steps are not required. Instead, the prenylated proteins (specifically Rab-family small GTPases) are directly targeted to their respective membranes by Rab escort proteins (REP) (Rilling et al., 1989; Boyartchuk et al., 1997; Rodriguez-Concepcion et al., 1999).

**Table 1:** Comparation of type I and type II protein prenylation.

Feathers	Type I	Type II
C-terminal conserved motif	CaaX(C = cysteine, a = aliphatic amino acid, X= variable amino acid)	CC(Di-cysteine motif, C = cysteine)
Representative protein families	Ras、Rho and related small GTPases	Rab small GTPases
Key catalytic enzyme	Farnesyltransferase(FTase) Geranylgeranyltransferase (GGTase-I)	Rab geranylgeranyltrans- ferase(Rab-GGTase)
Isoprenoid lipid donor	FPP GGPP	GGPP
Number of lipid chains	Single isoprenoid chain	Two geranylgeranyl chains
Post-prenylation processing steps	Protein cleavage(Carboxyl peptidases)、Carboxy-terminal methylation(Carboxyl methyl transferase)	Directly targeted to their respective membranes by Rab escort proteins (REP)

The initial step is catalyzed by prenyltransferases, which transfer the isoprenoid group to the substrate protein. Three distinct prenyltransferases recognize different C-terminal motifs: farnesyltransferase (FTase) and two types of geranylgeranyltransferases (GGTase-I and GGTase-II/Rab-GGTase) (Schafer and Rine, 1992). Both FTase and GGTase-I recognize a conserved C-terminal CaaX motif, where C is cysteine, a is typically an aliphatic amino acid, and X can be any residue but often determines enzyme specificity. When X is leucine, the protein is preferentially geranylgeranylated by GGTase-I; otherwise, it is typically farnesylated by FTase (Schafer and Rine, 1992; Griggs et al., 2010). In contrast, in TypeII modification, Rab-GGTase specifically modifies Rab-family small GTPases that contain C-terminal double-cysteine motifs such as XXCC, XCCX, CCXX, or XCXC. This enzyme attaches two geranylgeranyl groups, resulting in dual prenylation of the cysteine residues (Seabra et al., 1992). In this step, both FTase and GGTase-I function as heterodimeric enzymes. They share an identical α-subunit but possess distinct β-subunits, which determine their specificity for different substrate proteins (Schafer and Rine, 1992; Zhang and Casey, 1996). Both FTase and GGTase-I require Zn²^+^ for catalytic activity. The Zn²^+^ ion is coordinated at the interface of the α- and β-subunits, helping to form two key clefts: one for binding the protein substrate and another for accommodating the isoprenoid donor, such as farnesyl diphosphate (FPP) in the case of FTase (Reiss et al., 1992; Zhang and Casey, 1996; Park et al., 1997). Rab-GGTase is a multisubunit enzyme consisting of a catalytic heterodimer and an accessory component, named rab escort protein REP (Zhang and Casey, 1996).

Emerging evidence have revealed that protein prenylation is not strictly limited to proteins containing the canonical C-terminal CaaX motif. Emerging evidence indicates that proteins with CXX or CXXXX motifs at their C-termini can also be recognized and modified by farnesyltransferase or geranylgeranyltransferase, leading to successful protein prenylation (Blanden et al., 2018; Ashok et al., 2020). This expanded mode of modification is now referred to as atypical protein prenylation.

Following the attachment of an isoprenoid group to the cysteine within the CaaX motif, the second step entails the proteolytic cleavage of the “aaX” residues. This hydrolysis is catalyzed by specific ER membrane-associated zinc metalloproteases, which expose the C-terminal prenylated cysteine residue (Gao et al., 2009). In *Saccharomyces cerevisiae*, this proteolytic activity is mediated by the STE24 and RCE1 gene products (Schmidt et al., 1998). STE24 cleaves the “-aaX” motif from the yeast mating pheromone a-factor, whereas RCE1 cleaves it from both a-factor and Ras2 (Leung et al., 2001). The third step involves the methylation of the newly exposed cysteine residue by a membrane-associated, S-adenosylmethionine (SAM)-dependent isoprenylated cysteine carboxymethyl transferase (ICMT). This reaction yields a C-terminal cysteine methyl ester (Griggs et al., 2010; Yang et al., 2011). Notably, this methylation is reversible. Demethylation can be catalyzed by isoprenylcysteine carboxyl methyl esterases, such as ICME or PCME, which remove the methyl group from the prenylated cysteine (Huizinga et al., 2008).

## Reassessing the landscape of putative prenylated chloroplast proteins

The existence of prenylated chloroplast proteins has been known for decades. A seminal study by Parmryd et al, using *in vivo* labeling with [³H] mevalonate in spinach, identified approximately 20 prenylated proteins (35–46 kDa), the vast majority of which were membrane-associated. About 40% localized to the chloroplast outer envelope, with the remainder found in thylakoid or inner envelope membranes; no stromal proteins were found to be modified (Parmryd et al., 1997). Furthermore, prenylated thylakoid proteins were primarily detected as pigment-protein complexes (Parmryd et al., 1997). These findings cemented the early view that chloroplast prenylation predominantly targets membrane proteins—a notion that appeared to contrast with systems in Yeast and Mammals, where a significant proportion of prenylated proteins are soluble (Maltese, 1990; Hildebrandt et al., 2016). Nevertheless, these biochemical characterizations were exclusively performed in spinach and have yet to be confirmed in other plant species.

However, contemporary proteomic analyses challenge this paradigm. Screening of databases such as PPDB reveals that among 2,495 chloroplast-localized proteins in Arabidopsis(**Table 2**), 64 possess a C-terminal CaaX motif—16 with a canonical sequence and 48 with atypical CXXX(n) variants, marking them as potential prenylation substrates. Intriguingly, only a small fraction of these candidates are membrane proteins (six thylakoid and four envelope-associated), while the majority are soluble stromal proteins. Functional annotation of these soluble candidates links them to chloroplast maintenance and metabolism, with the cytokinin synthase IPT3 (which carries a “CLVA” motif) representing a experimentally validated example (discussed later). Although further validation is needed, these observations suggest that the scope of prenylated chloroplast proteins likely extends far beyond membrane-associated clients.

**Table 2:** Analysis of the CaaX motif in chloroplast proteins of Arabidopsis.

Numbers	Gene	Protein	Localization	CaaX	Size/kDa	Function
Canonical “CaaX” motif						
1	At1g07160^#^	PP2C	Plastid	CHLF	40	Protein phosphatase 2C family protein
2	At1g12250	Pentapeptide repeat protein	Thylakoid membrane	CDVK	30	Thylakoid biogenesis.
3	At1g55370.1	NDF5	Thylakoid	CGFI	40	NDH-dependent cyclic electron flow 5
4	At1g73540	NUDT21	Plastid	CQTQ	18	Inositol pyrophosphate degradation
5	At1g78010.1^#^	TRME	Plastid	CIGK	61	tRNA modification GTPase
6	At2g21385.4	BFA3	Plastid stroma	CFSS	38	Biogenesis factors required for ATP synthase 3
7	At2g31690	DALL5	Plastid.	CNIF	46	Degradation of triacylglycerol
8	At3g01510	LSF1	Plastid stroma	CPKS	65	A putative phosphatase
9	At3g12080.2	EMB2738	Plastid	CTQQ	73	Putative plastid-targeted GTP-binding protein
10	At3g15690.1	BLP2	Envelope-inner-peripheral-stromal-side	CRIQ	26	Putative biotin carboxyl carrier protein
11	At3g26690	NUDX13	Plastid	CFVV	23	Mitochondrial Nudix hydrolase
12	At3g47490^#^	HNH endonuclease	Plastid	CRIQ	26	Endonuclease activity
13	At3g63110	IPT3	Plastid	CLVA	38	Isopentenyltransferase 3
14	At5g21222^#^	Protein kinase family protein	Plastid	CKFM	93	Protein kinase activity
15	At5g42765	Plasma membrane fusion protein	Thylakoid	CLEI	25	Unknown
16	Atcg00770★	S8 ribosome	Plastid ribosome	CYIW	15	Ribosomal protein S8
Non-canonical “CaaX” motif						
17	At1g06730.1	PNK1	Plastid	CVKASS	53	Plastid nucleoside kinase
18	At1g08070	OTP82	Plastid	CNDYW	84	Chloroplast RNA editing factor
19	At1g11290	CRR22	Plastid	CGDYW	91	Endonuclease activity
20	At1g12800	SDP	Plastid stroma	CTNRVE	86	RNA binding protein
21	At1g15510	ECB2	Plastid stroma	CGD	98	Pentatricopeptide repeat protein
22	At1g15730	ZNG2A2	Plastid	CLI	50	Similar to ZNG1 nucleotide- dependent metallochaperone
23	At1g18060	FBN-like	Thylakoid-peripheral-stromal-side	CRRIAS	25	Microbial collagenase
24	At1g19720	PPR-like	Plastid	CKDYW	101	Pentatricopeptide repeat superfamily protein
25	At1g35250	ALT2	Plastid	CQHLVD	21	Acyl-lipid thioesterase 2
26	At1g42970	GAPB	Plastid stroma	CKVYD	48	Glyceraldehyde-3-phosphate dehydrogenase B subunit
27	At1g48850.2	EMB1144	Plastid stroma	CAWSS	41	Flavoenzyme-encoding gene
	At1g48850.3					
28	At1g51400	PsbTn-2	Thylakoid-integral	CRN	11	Photosystem II 5 kD protein
29	At1g52590	Putative thiol-disulfide oxidoreductase DCC	Plastoglobules	CEL	20	Unknow
30	At1g59720	CRR28	Plastid stroma	CLDYW	72	Endonuclease activity
31	At1g64970	G-TMT	Plastid envelope	CQKPL	38	Gamma-tocopherol methyltransferase
32	At1g69740	ALAD1	Plastid stroma	CGEKR	47	5-aminolevulinate dehydratase
33	At1g79050.2	RECA1	Plastid stroma	CST	37	RecA DNA recombination family protein
34	At1g80480	PTAC17	Plastid stroma	CLI	50	Plastid transcriptionally active 17
35	At2g02980	OTP85	Plastid	CGDFW	68	Chloroplast RNA editing factor
36	At2g10940	Bifunctional inhibitor/lipid-transfer protein/seed storage 2S albumin superfamily protein	Plastid	CSI	30	Lipid-transfer
37	At2g11810.2	MGDC	Outer Envelope	CIITKH	42	Monogalactosyl diacylglycerol synthase 3
38	At2g15690	DYW2	Plastid/Mitochondria	CGDYW	66	Atypical PPR-DYW protein
39	At2g20920	CRS	Plastid	CLR	30	Plant senescence
40	At2g24120	SCA3	Plastid/Mitochondria	CFH	101	DNA/RNA polymerases superfamily protein
41	At2g29760	OTP81	Plastid	CNDFW	83	Chloroplast RNA editing factor
42	At2g36000.2	MTERF3	Plastid	CNE	36	Mitochondrial transcription termination factor
43	At2g37240	Thioredoxin superfamily protein	Plastid stroma	CA	27	Thioredoxin-like/alkyl hydroperoxide reductase
44	At2g39730.1	RCA	Plastid stroma	CVYNF	52	Rubisco activase
45	At3g01370	CFM2	Plastid stroma	CGL	114	CFM2 splice factor
46	At3g04000	CHLADR	Plastid	CLL	28	Chloroplast aldehyde reductase
47	At3g11560	LETM1-like protein	Plastid envelope	CHFLF	70	Unknow
48	At3g20390	RIDA	Plastid stroma	CIATL	20	Reactive intermediate deaminase A
49	At3g21055	PSBTN	Thylakoid-integral	CRY	11	Photosystem II subunit T
50	At3g23940	DHAD	Plastid stroma	CVTDE	65	Dihydroxyacid dehydratase
51	At3g46790	CRR2	Plastid stroma	CGDYW	74	A member of plant combinatorial and modular protein family
52	At3g57430	OTP84	Plastid	CGDYW	99	Chloroplast RNA editing factor
53	At4g01030	PPR-like	Plastid	CNDSW	86	Pentatricopeptide (PPR) repeat-containing protein
54	At4g09620	MTERF12	Plastid stroma	CWVRF	24	Mitochondrial transcription termination factor
55	At4g12470^#^	AZI1	Outer Envelope	CA	17	Azelaic acid induced 1
56	At4g18750	DOT4	Plastid	CRGFW	98	Pentatricopeptide repeat (PPR) superfamily protein
57	At4g22890.2	PGRL1A	Thylakoid-integral	CTK	34	Photosynthetic electron transport in photosystem I
58	At4g31780.1	MGDA	Inner Envelope	CTA	41	A-type monogalactosyldiacylglycerol synthase
	At4g31780.2				56	
59	At5g23040	CDF1	Plastid envelope	CTFLK	29	Cell growth defect factor 1
60	At5g35210.2	PTM	Outer Envelope	CREWQ	172	PHD type transcription factor with transmembrane domains
61	At5g52970	ECM1	Thylakoid-peripheral-lumenal-side	CAEIQ	25	Thylakoid lumen 15.0 kDa protein
62	At5g67030.2	ABA1	Thylakoid-integral	CDR	67	Abscisic acid biosynthetic
63	Atcg00065★	RPS12A	Plastid ribosome	CTRVY	4	Ribosomal protein s1
64	Atcg00180★	RPOC1	Plastid nucleoid	CSYDT	79	RNA polymerase beta’ subunit-1

Proteins marked with stars are encoded by the chloroplast genome. Proteins possessed a clear signal peptide predicted by TargetP and had been experimentally confirmed to localize in the chloroplasts (see PPDB database: http://ppdb.tc.cornell.edu/) were listed. Localization of five proteins remains controversial based on current proteomic data; these entries have now been marked with a hash key. Entries marked with asterisks indicate that they are derived from chloroplast genomes.

How then can we reconcile the discrepancy regarding the scope of prenylated chloroplast proteins between predicted CaaX proteins and early *in vivo labeling* data? This may relate to phytol, another product of the plastidial MEP pathway. Serving as the prenyl side chain of chlorophyll, phytol mediates its association with chlorophyll-binding proteins in the thylakoid membrane. The *in vivo* [³H]mevalonate labeling used in earlier studies cannot distinguish between modifications by farnesol/geranylgeraniol and those associated with phytol s (Parmryd et al., 1999).This limitation might explain why early *in vivo* labeling assays overwhelmingly detected membrane-associated signals and effectively “masked” the less abundant farnesyl/geranylgeranyl modifications. Collectively, these insights lead us to a revised model: both membrane-associated and soluble chloroplast proteins can be prenylated, with plastid-encoded proteins predominantly undergoing phytolation and nuclear-encoded proteins primarily modified by farnesol or geranylgeraniol. This new perspective fundamentally expands and refines our understanding of the putative substrate repertoire for chloroplast prenylation.

The analytical characterization of protein prenylation is technically challenging, and to date, only a limited number of CaaX-containing proteins have been experimentally confirmed to undergo this modification (Chevalier et al., 2025). This suggests that the presence of a CaaX motif is not an absolute guarantee of prenylation. Over the years, several computational tools have been developed to identify potential prenylation substrates by evaluating the likelihood that a given protein is targeted by prenyltransferases. The Prenylation Prediction Suite (PrePS) is one such method that models substrate-enzyme interactions based on the refinement of recognition motifs for each prenyltransferase (Maurer-Stroh and Eisenhaber, 2005). Using PrePS, a considerable number of Arabidopsis proteins have been predicted to serve as substrates for prenyltransferases (encompassing both Type I and Type II protein prenylation) (Chevalier et al., 2025). Our analysis revealed that nine of these putative prenyltransferase substrates are predicted to localize to chloroplasts (**Table 3**). Surprisingly, only three of these 9 proteins harbor CaaX-related motifs, suggesting that some of them might not be subjected to post- prenylation maturation. These independent prediction results further support the hypothesis that certain chloroplast proteins undergo prenylation, although further experimental evidence is still required.

**Table 3:** Putative prenyltransferase substrates in chloroplasts predicted by PrePS.

Gene	PPDB	Target P
AT3g12080	**+**	**+**
At3g15690	**+**	**+**
AT3g63110	**+**	
At1g05810		**+**
At1g34010		**+**
At2g18196		**+**
At2g44690		**+**
At5g11840		**+**
At4g10465		**+**

The putative protein substrates containing CaaX-related motifs are underlined and their chloroplast localizations predicted by Target P and/or verified by the proteomic evidence from PPDB (Plant Proteome Database).

## A putative unique pathway for chloroplast protein prenylation

Nuclear-encoded chloroplast proteins are synthesized in the cytosol as precursors and imported into chloroplasts via the TOC-TIC complex (Jarvis and Soll, 2001; Soll and Schleiff, 2004). Given that isoprenoid precursors are synthesized in both the cytosol/peroxisomes and chloroplasts (Lichtenthaler et al., 1997; Sapir-Mir et al., 2008), the canonical prenylation of nuclear-encoded proteins could, in principle, occur either in the cytosol (on precursor forms) or within chloroplasts (on mature proteins). In Arabidopsis, the identified farnesyltransferase (FTase) and geranylgeranyltransferase I (GGTase-I) subunits—FTA/PLP (shared α-subunit) and ERA1 (FTase β-subunit) (Cutler et al., 1996; Running et al., 2004)—along with the Rab-GGTase subunits (RGTA1/RGTA2 and RGTB1/RGTB2) (Shi et al., 2016), have not been reported to localize to chloroplasts (**Table 4**). This suggests that the initial prenylation step for nuclear-encoded proteins most likely occurs in the cytosol, although a chloroplast-localized activity cannot be entirely ruled out.

**Table 4:** Putative protein prenylation components in Arabidopsis.

Gene	Protein	Encoding subunit	Localization (TAIR or publication)
Farnesyltransferases (PFT)、Geranylgeranyltransferase (PGGT1/GGTase-I)			
At3g59380	FTA/PFT-α/PGGT1-α/PLP	Common α subunit of PFT and PGGT1	Cytoplasm
At5g40280	ERA1/PFT-β	β subunit of PFT	Mitochondria/Cytoplasm
At2g39550	PGGT1-β/GGB	β subunit of PGGT1	Cytoplasm
Rab geranylgeranyltransferase (GGTase-II/Rab-GGTase)			
At4g24490	RGTA1	α subunit of GGTase-II	Cytoplasm
At5g41820	RGTA2	α subunit of GGTase-II	Cytoplasm
At5g12210	RGTB1	β subunit of GGTase-II	Cytoplasm
At3g12070	RGTB2	β subunit of GGTase-II	Cytoplasm
At1G10095	Unknown	Unknown	Cytoplasm
CaaX proteases			
At4g01320	STE24		Endoplasmic reticulum (Bracha-Drori et al., 2008)
At2g36305	RCE1		Endoplasmic reticulum (Bracha-Drori et al., 2008)
At5g60750	SCO4		Chloroplast (Albrecht-Borth et al., 2013)
AT1g14270	Unknow		Chloroplast
At2g20725	Unknow		Chloroplast
Isoprenylcysteine methyltransferase			
At5g23320	ICMTA		Endoplasmic reticulum (Bracha-Drori et al., 2008)
At5g08335	ICMTB		Endoplasmic reticulum (Bracha-Drori et al., 2008)
At5g59500	TAM46		Chloroplast envelop (Liang et al., 2024)

If cytosolic prenylation does occur, the subsequent processing steps—proteolytic cleavage and methylation—present an intriguing topological question. One possibility is that these steps take place in the endoplasmic reticulum (ER), with the fully processed protein then trafficked to chloroplasts, potentially via the secretory pathway. However, only a limited number of glycosylated chloroplast proteins have been shown to utilize this route (Nanjo et al., 2006; Radhamony and Theg, 2006). Furthermore, the membrane association acquired during prenylation processing makes it difficult to explain how such modified proteins could be efficiently targeted to internal chloroplast membranes such as thylakoids. Therefore, if this pathway exists, it is unlikely to be the primary mechanism.

Notably, the Arabidopsis genome encodes five putative CaaX proteases (**Table 4**). While FACE2/RCE1 and STE24 are canonical, ER-localized proteases (Bracha-Drori et al., 2008), the other three are intriguingly predicted to be chloroplast-localized membrane proteins. Among them, AT5G60750 (SCO4) contains a chloroplast transit peptide and has been experimentally confirmed to reside in chloroplasts. Although evolutionarily distinct from canonical CaaX proteases, SCO4 expression partially rescues the temperature sensitivity of yeast *rce1*Δ mutants, indicating retained protease activity in a heterologous system (Albrecht-Borth et al., 2013). This positions SCO4 as a plausible candidate for conducting the proteolytic cleavage of prenylated proteins in chloroplasts. Intriguingly, SCO4-GFP localizes in punctate signals on the chloroplast surface (Albrecht-Borth et al., 2013), reminiscent of outer envelope membrane proteins (Machettira et al., 2011), suggesting it may be anchored at this interface. Collectively, these observations support a model in which nucleus-encoded proteins are prenylated in the cytosol and subsequently undergo proteolytic cleavage at the chloroplast outer envelope, possibly mediated by SCO4. In addition to SCO4, two other putative CaaX proteases are predicted to localize in chloroplasts, broadening the pool of potential candidates.

If proteolytic cleavage occurs at the outer envelope, the final methylation step might also take place there. While the known Arabidopsis methyltransferases ICMTA and ICMTB are ER-localized (Bracha-Drori et al., 2008), proteomic data highlight another putative methyltransferase, AT5G59500 (TAM46). This protein possesses seven transmembrane domains and localizes to the chloroplast envelope. Although not a core component of the TOC-TIC complex, TAM46 interacts with the Ycf2-FtsHi complex, which is involved in protein import into the stroma (Liang et al., 2024). We noted that lipid molecules were also identified within Ycf2/FtsHi complex, and some of its components are subject to post-translational modifications (Liang et al., 2024). Although this evidence is highly indirect, it raises the intriguing possibility that TAM46 and its associated complex might participate in lipidation-related PTMs.

**Figure 1:**
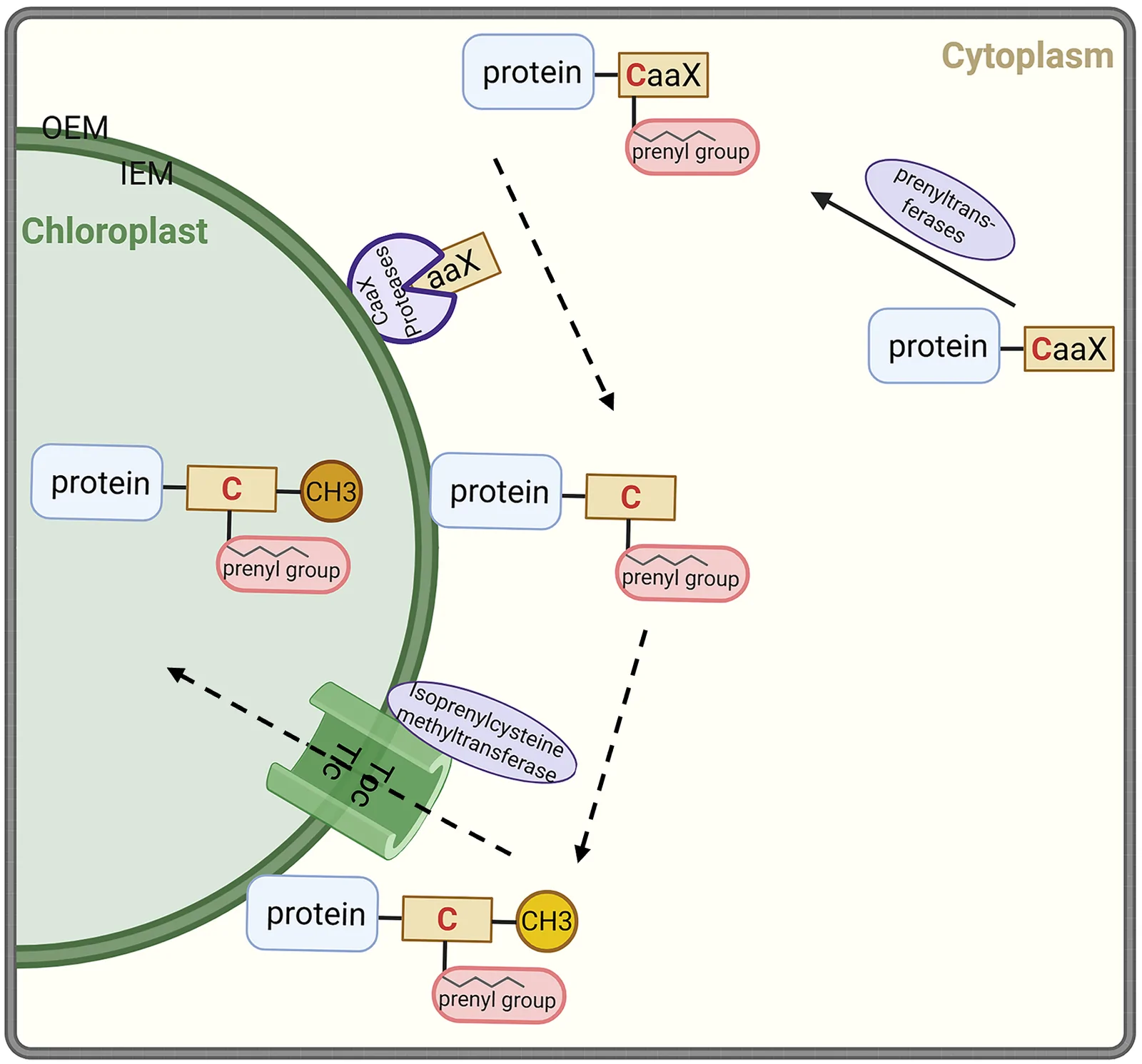
Working model diagram of prenylation modification of chloroplast proteins. Chloroplast proteins encoded by nuclear genes undergo the first step of isoprenylation modification in the cytoplasm—the addition of an isopentenyl group to the C-terminal cysteine residue; subsequently, the aaX amino acids are removed by CaaX proteases located in the outer envelope membrane of chloroplasts; finally, the methylation step is completed on the outer envelope membrane of chloroplasts under the action of isoprenyl methyltransferases. Ultimately, the isoprenylated chloroplast proteins translocate into chloroplasts via the Toc-Tic complex to exert their biological functions. OEM stands for the outer envelope membrane of chloroplasts, and IEM stands for the inner envelope membrane of chloroplasts. The hypothetical steps of this pathway are indicated by dotted lines. In the future, the validity of our model can be verified through a combination of biochemical and genetic approaches. For instance, metabolic labeling with modified isoprenoid analogs (e.g., azido-geranyl diphosphate), combined with subcellular fractionation, would allow for the specific detection of prenylated chloroplast precursors in the cytosol and at the envelope. Furthermore, genetic disruption of cytosolic prenyltransferases or candidate envelope processing enzymes is expected to result in distinct defects in the import of CaaX-motif-containing chloroplast proteins, which can be quantitatively assessed via *in vitro* import assays. Finally, site-directed mutagenesis of the catalytic residues of candidate CaaX proteases (e.g., SCO4) should lead to the accumulation of prenylated precursors at the chloroplast envelope and impair their proper import, a phenotype that can be monitored using fluorescent protein fusions.

In light of these findings that the presence of putative CaaX proteases and methyltransferases in the chloroplast envelope, we propose a novel pathway in which the proteolytic and methylation steps occur at the envelope rather than the ER (**Figure 1**). This model is consistent with early biochemical evidence showing that a significant fraction of prenylated polypeptides is detected at envelope membranes (Parmryd et al., 1997). While direct experimental validation is still needed, this hypothesis offers a coherent framework for reconciling the cytosolic origin of prenylation with the chloroplast destination of its substrates. Nevertheless, until now, SCO4 and TAM46 are only two candidate proteins involved in this process and direct biochemical evidence is still absent.

## Potential integration of chloroplast protein prenylation into protein targeting machinery

Protein targeting to chloroplasts involves multiple pathways. For interior chloroplast compartments—such as the stroma, thylakoid membrane, and inner envelope—an N-terminal transit peptide is generally sufficient to direct proteins from the cytosol (Bruce, 2001). In such cases, C-terminal modifications like prenylation would theoretically play little role in initial targeting. In contrast, most outer envelope membrane (OEM) proteins are encoded without a cleavable transit peptide, implying distinct targeting mechanisms (Wu and Ko, 1993; Jarvis, 2008; Armbruster et al., 2009). For instance, studies on Toc64 have shown that both its transmembrane domain and a C-terminal lysine-rich flanking region are necessary for correct OEM localization, underscoring the potential importance of C-terminal features in outer envelope targeting (Sohrt and Soll, 2000; Lee et al., 2004).

This combination of transmembrane domain and C-terminal extension may represent a broader mechanism for determining localization to the endoplasmic reticulum or endosymbiotic organelles. However, this model cannot explain the targeting of OEM proteins that lack transmembrane domains. Interestingly, several putative prenylated OEM proteins, such as MDG3 (Awai et al., 2001), lack both a transit peptide and transmembrane domains. This observation leads us to hypothesize that prenylation may provide an alternative targeting mechanism for such proteins: by enhancing their membrane affinity, prenylation could facilitate their anchorage to the outer envelope and prevent their relocation to the cytosol.

A key requirement for post-translational protein translocation across biological membranes, including those of chloroplasts and mitochondria, is that substrate proteins must be at least partially unfolded to traverse the translocation machinery (Eilers and Schatz, 1986). Acidic phospholipids in the outer mitochondrial membrane, for example, have been shown to mediate partial unfolding of precursor proteins such as the artificial fusion protein pCOX IV–dihydrofolate reductase (Endo et al., 1989). In chloroplasts, attempts to inhibit import of dihydrofolate reductase fusion proteins containing a chloroplast transit peptide were unsuccessful, suggesting the presence of strong unfolding activity at the chloroplast envelope (Guéra et al., 1993; America et al., 1994). Within this framework, we hypothesize that C-terminal prenylation of precursor proteins could influence this unfolding activity, thereby modulating import efficiency—not only for outer envelope proteins but also for those destined for the inner envelope, stroma, or thylakoids. While the transit peptide (not the C-terminus) serves as the primary recognition signal for TOC complex receptors, the physicochemical changes induced by prenylation may still affect the translocation process (Becker et al., 2004; Bédard and Jarvis, 2005). However, the effect of prenylation is not uniformly pro-unfolding; in some contexts, a C-terminal farnesyl moiety can enhance protein folding rather than destabilization (Pylypenko et al., 2008). For instance, prenylation-mediated lipid binding at a hydrophobic pocket reduces the conformational flexibility of Ykt6, thereby maintaining its folded state (Weng et al., 2015). Under such conditions, prenylation could impair rather than facilitate translocation. A illustrative example is the IPT3 protein: when farnesylated, it localizes to the nucleus (Kasahara et al., 2004); when unfarnesylated, it is redirected to the chloroplast stroma (Galichet et al., 2008). This case supports the view that prenylation may serve as a regulatory switch, not only influencing the efficiency of chloroplast import but also contributing to the alternative localization of dual-targeted chloroplast proteins.

Beyond its potential influence on the folding and unfolding states of chloroplast protein precursors, prenylation may regulate chloroplast protein import through alternative mechanisms. For instance, the prenylation of precursor proteins could promote their association with cytosolic factors, effectively retaining them in the cytosol and thereby attenuating their trafficking to the chloroplast envelope. Notably, the putative prenylation component Tam46 has also been identified as an integral part of the chloroplast protein import motor (the FtsHi-YCF2 complex) (Liang et al., 2024). This dual involvement suggests a more direct mechanistic link, implying that prenylation might directly modulate the efficiency of the translocation machinery itself.

## Concluding remarks and future perspectives

Despite earlier reports documenting the existence of prenylated chloroplast proteins, the full scope of this modification, including the specific proteins modified and the precise subcellular sites where prenylation occurs, remains poorly understood. In this review, we have proposed that the majority of prenylated chloroplast proteins are nuclear-encoded. Rather than supporting a fully autonomous chloroplast pathway, we suggest a model in which prenylation involves coordinated activity between the cytosol and the chloroplast envelope compartments that are physically linked (**Figure 1**). It should be highlighted that this pathway is only a hypothesis and an organelle-autonomous prenylation pathway in plastids could not been excluded completely.

In **Table 2**, we list 64 putative chloroplast proteins harboring C-terminal CaaX-related motifs in *Arabidopsis thaliana*. To the best of our knowledge, IPT3 remains the only one experimentally confirmed to be prenylated *in vivo*. It is important to note that the mere presence of such motifs does not guarantee that these proteins are necessarily prenylated. Furthermore, w we acknowledge the alternative possibility that certain prenylated proteins might localize to chloroplasts despite not being bioinformatically predicted to do so. Therefore, a comprehensive understanding of the chloroplast prenylome landscape requires further experimental investigation.

Advances in cell biology, omics, and imaging have increasingly revealed previously unappreciated ER-chloroplast membrane contact sites (MCSs) (Depaepe and Munné-Bosch, 2026). These contact sites act as molecular crossroads for plastid lipid metabolism. Given that prenylation is inherently a lipid-based modification and its subsequent processing might occur at the chloroplast envelope, it is highly logical to posit that ER-chloroplast MCSs could serve as critical hubs for the transfer or modification of prenylated precursors. Although direct experimental evidence is currently lacking, investigating the involvement of MCSs represents a highly relevant and promising perspective for understanding the mechanisms of chloroplast protein prenylation.

Moving forward, several research directions appear particularly promising. First, identifying the genuine enzymatic components responsible for the proteolytic cleavage and methylation of prenylated chloroplast proteins represents an essential next step. Second, the application of emerging tools, such as dual chemical probes for prenylation (Storck et al., 2019), could enable proteome-wide quantitative analysis and reveal the dynamics of this modification in chloroplasts. Finally, exploring the physiological significance of chloroplast protein prenylation, especially its potential role in regulating protein targeting, import, and the plasticity of the chloroplast proteome, will be critical to understanding its functional impact on chloroplast biology and plant physiology.
